# Reductive Reactivity of Iron(III) Oxides in the East China Sea Sediments: Characterization by Selective Extraction and Kinetic Dissolution

**DOI:** 10.1371/journal.pone.0080367

**Published:** 2013-11-15

**Authors:** Liang-Jin Chen, Mao-Xu Zhu, Gui-Peng Yang, Xiang-Li Huang

**Affiliations:** Key Laboratory of Marine Chemistry Theory and Technology, Ministry of Education, College of Chemistry and Chemical Engineering, Ocean University of China, Qingdao, China; University of California, Merced, United States of America

## Abstract

Reactive Fe(III) oxides in gravity-core sediments collected from the East China Sea inner shelf were quantified by using three selective extractions (acidic hydroxylamine, acidic oxalate, bicarbonate-citrate buffered sodium dithionite). Also the reactivity of Fe(III) oxides in the sediments was characterized by kinetic dissolution using ascorbic acid as reductant at pH 3.0 and 7.5 in combination with the reactive continuum model. Three parameters derived from the kinetic method: *m*_0_ (theoretical initial amount of ascorbate-reducible Fe(III) oxides), *k*′ (rate constant) and *γ* (heterogeneity of reactivity), enable a quantitative characterization of Fe(III) oxide reactivity in a standardized way. Amorphous Fe(III) oxides quantified by acidic hydroxylamine extraction were quickly consumed in the uppermost layer during early diagenesis but were not depleted over the upper 100 cm depth. The total amounts of amorphous and poorly crystalline Fe(III) oxides are highly available for efficient buffering of dissolved sulfide. As indicated by the *m*_0_, *k*′ and *γ*, the surface sediments always have the maximum content, reactivity and heterogeneity of reactive Fe(III) oxides, while the three parameters simultaneously downcore decrease, much more quickly in the upper layer than at depth. Albeit being within a small range (within one order of magnitude) of the initial rates among sediments at different depths, incongruent dissolution could result in huge discrepancies of the later dissolution rates due to differentiating heterogeneity, which cannot be revealed by selective extraction. A strong linear correlation of the *m*_0_ at pH 3.0 with the dithionite-extractable Fe(III) suggests that the *m*_0_ may represent Fe(III) oxide assemblages spanning amorphous and crystalline Fe(III) oxides. Maximum microbially available Fe(III) predicted by the *m*_0_ at pH 7.5 may include both amorphous and a fraction of other less reactive Fe(III) phases.

## Introduction

Iron (Fe) is the most abundant, redox-sensitive element on earth’s surface. Its redox cycling in marine sediments has a profound influence on cycling and fate of carbon, sulfur, phosphorus, and a variety of trace elements [1]–[3]. In oxic marine sediments, secondary Fe phases (from weathering of Fe-bearing primary minerals) occur dominantly as Fe(III) oxides, hydroxides, and oxyhydroxides (collectively hereafter referred to as Fe(III) oxides) with a wide spectrum of mineralogy, crystallinity, morphology and chemical compositions. Therefore they usually display quite variable adsorption affinity and reduction reactivity [4]–[6]. For example, reactive Fe(III) oxides serve as an important sink for a large number of dissolved species, particularly phosphorus, arsenic and heavy metals through surface adsorption and incorporation [7], [8], while their reductive dissolution in anoxic environments results in release of these elements into porewater and thus exerts a crucial control on benthic fluxes of these elements [8]–[10].

Chemical and microbial reductions are two competing mechanisms of Fe(III) oxide dissolution [11]–[13]. In organic matter (OM)-rich marine sediments where dissolved sulfide is readily available via sulfate reduction, chemical reduction of Fe(III) oxides by dissolved sulfide generally dominates over the microbial pathways since the former is thermodynamically more favored than the latter, whereas microbial Fe(III) reduction is more important in some other marine sediments where reactive Fe(III) is abundant and/or OM flux to the seabed is low to intermediate [1], [13]–[17]. Rates of both chemical and microbial Fe(III) reductions are strongly dependent on the reactivity of Fe(III) oxides [4], [18], [19]. Thus quantitative characterization of Fe(III) oxide reactivity is of significance for understanding Fe cycles and its influences on diagenesis of many other interlinked elements.

Selective extraction and kinetic dissolution are two commonly used methods to characterize relative reactivity of various Fe(III) oxides. Sequential/selective extractions, which are initially for Fe speciation, can be used to deduce relative reactivity of Fe(III) oxides based on specific extractants used. For example, the refined sequential extraction procedure of Poulton and Canfield [20] can be used to discriminate easily reducible Fe(III) oxides (Fe_ox1_) (mainly amorphous and poorly crystalline Fe(III) oxides such as ferrihydrite and lepodocrocite), reducible Fe(III) oxides (Fe_ox2_) (mainly goethite, akaganéite and hematite), magnetite, and poorly reactive silicate Fe pool. One disadvantage of the extraction methods is that they are empirically defined and nonmineral specific, and that Fe(III) oxides in a single pool are assumed to be chemically homogeneous. Actually, each extraction step may describe a heterogeneous group of minerals with a wide range of reactivity as a single pool [21].

Postma [22] introduced a more quantitative kinetic method to describe the reactivity of Fe(III) oxide assemblages, which could offer a more detailed and nuanced picture of composition and reactivity of Fe(III) oxides in comparison with the conventional chemical extractions. Reductive reactivity of natural Fe(III) oxide assemblages with variable crystallinity and surface properties can be described by a reactive continuum, and a dissolution rate law of the Fe(III) oxide assemblages can be expressed as [22], [23]:
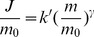
(1)where *J* is the reduction rate (μmol s^−1^), *k*′ is the apparent rate constant (s^−1^), which can be used as a measure of reductive reactivity, *m*_0_ is the theoretical initial mass of the reactive Fe(III) oxides, *m* is the remaining mass at a given time *t*, and *γ* is the apparent reaction order. Predicted *γ* value for ideal dissolving spheres or cubes is 2/3 [22]. In natural Fe(III) oxide assemblages *γ* is the function of crystal morphology, particle size distribution and reactive site density, and hence is a measure of the heterogeneity of the Fe(III) oxide assemblages undergoing dissolution [22], [23]. Alternatively, Eq. (1) can be expressed in a logarithmic form:
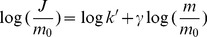
(2)that provides straight lines with slope γ and intercept log k′. Integrating Eq. (1) for γ ≠ 1 yields:



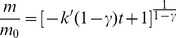
(3)Eq. (3) is based on the fraction of undissolved Fe(III) oxide (*m*/*m*_0_) but the experimental data yield the dissolved amount *m_t_* at time *t*. The amount of *m* can be obtained by subtracting *m_t_* from *m*_0_. For the convenience of data processing, Eq. (3) is converted to Eq. (4):
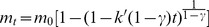
(4)


Optimized values of *m*_0_, *k*′ and *γ* can be simultaneously determined by fitting time-dependent dissolution data to Eq. (4) using a nonlinear least square procedure.

Time-dependent reductive dissolution of Fe(III) oxides by applying the kinetic method above has been commonly recorded at pH 3.0 using ascorbic acid as a reductant [21]–[27]. The kinetic method offers a standardized way to compare intrinsic reactivity of Fe(III) oxides, however, how to link the experimental measurement to the *in situ* reactivity of Fe(III) oxide reduction in natural environments remains to be solved [21]. The kinetic investigation has also been done in citrate-bicarbonate (CB) buffered ascorbate solution at pH 7.5 [24], [28], [29]. In comparison with the investigation at pH 3.0, the CB-buffered ascorbate is more selective to the most reactive fraction of Fe(III) oxides and thus offers another standard to characterize intrinsic reactivity of Fe(III) oxides. More significantly, kinetic parameters derived at pH 7.5 bear specific microbial implications: *k*′ obtained at this pH, according to Hyacinthe et al. [24], can be used as a forecast proxy of microbial reactivity of Fe(III) oxides and *m*_0_ as a maximum estimate of bioavailable Fe(III) present in sediments, even though, on average, bacteria can reduce only 65% of the estimated reactive Fe(III) because of the inaccessibility of a fraction of the Fe(III) pool to iron reducers due to physical occlusion [19], [30].

In this contribution, selective extraction and kinetic dissolution were combined to investigate reductive reactivity of Fe(III) oxides in one gravity core collected from the East China Sea (ECS) inner shelf. The objectives of this study are: (1) to reveal the effects of early diagenesis on reductive reactivity of Fe(III) oxides based on depth-dependent variability of the three kinetic parameters, *m*_0_, *k*′ and *γ*; (2) to compare selective extraction and kinetic dissolution with regard to the reactivity of Fe(III) oxides.

## Materials and Methods

### Ethics statement

No official permission is required for the field studies because the study site is accessible to every Chinese citizen for scientific purposes and no protected species were sampled.

### Study area and sampling

**Figure 1:**
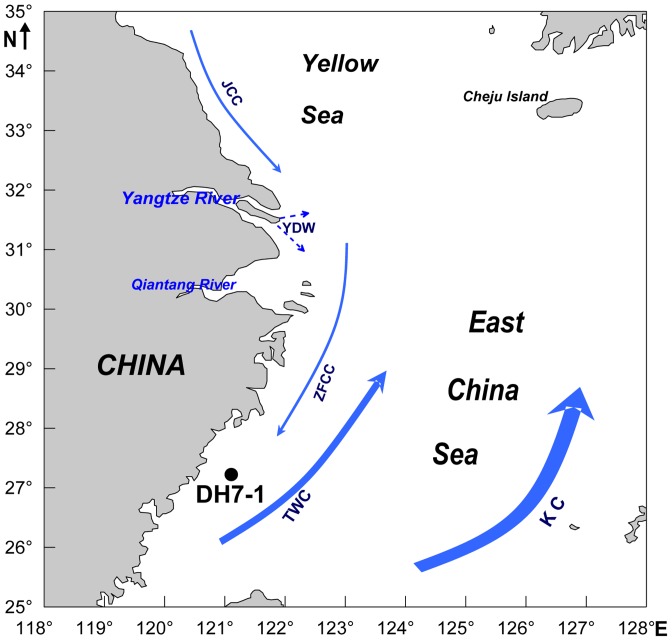
Regional ocean circulation patterns and sampling station in the East China Sea. JCC: Jiangsu Coastal Current; KC: Kuroshio Current; TWC: Taiwan Warm Current; YDW: Yangtze Diluted Water; ZFCC: Zhejiang-Fujian Coastal Current.

The ECS is one of the world’s largest shelf seas (Fig. 1). Annually it receives 5×10^8^ tonnes of terrestrial particulates [31], [32], mainly from the Yangtze River, the third longest river in the world. A large amount of fine-grained particulates is transported southward along the coast driven by the Jiangsu and Zhejiang-Fujian Coastal Currents, and trapped in the inner shelf by the blocking of the northward Warm Taiwan Current offshore, developing an elongated inner-shelf mud wedge from the Yangtze River mouth into the Taiwan Strait [33]. The shelf sediment, particularly the inner shelf mud, is an important sink for Fe [34], [35]. Chemical reduction of reactive Fe(III) in the sediment plays an important role in sulfur diagenetic cycling [35]–[38], while microbial Fe(III) reduction is expected to be also important in OM decomposition [35], [39]–[41].

One gravity core was collected at site DH7-1 (121.10°E, 27.23°N, water depth 30 m) during the cruise from October 11th to 28th, 2011 (Fig. 1). Upon retrieval, the gravity core (length 168 cm) was immediately sectioned at 3−5-cm intervals over the upper 45 cm and 10-cm interval below this depth in N_2_ atmosphere. All the sub-samples were sealed in zip-lock plastic bags under N_2_ atmosphere and frozen at −18°C until further handling.

### Selective extractions

Accurately preweighed frozen sediments in duplicate were dried at 105°C until constant weight for determination of wet/dry weight ratios, which were used to express contents of extracted solid-phase Fe in µmol per gram dry sediment (µmol g^−1^). To quantify Fe(III) oxide pools with different reactivity, three selective extractions were used for speciation of solid-phase Fe in 10 selected layers of the core (see Table 1 for the details). The extractions were carried out at room temperature, unless otherwise stated.

**Table 1:** Depth distributions of various solid-phase iron pools in gravity core DH7-1 of the East China Sea (unit: µmol Fe per gram dry sediment).

Depth (cm)	Acetate-Fe(II)	SD	Oxalate-Fe(III)	SD	BCD-Fe(III)	SD	Hydroxylamine-Fe(III)	SD	Magnetite	SD
1.5	40.1	0.05	132	16.5	191	0.54	16.7	0.02	41.7	3.07
5.5	40.6	0.26	138	8.09	142	0.07	12.1	0.17	40.3	4.26
10.5	41.2	0.87	131	15.0	154	0.87	8.88	0.83	36.2	2.46
15.5	42.0	0.31	124	2.05	189	8.55	7.70	0.19	39.4	6.29
30.5	42.4	0.83	124	10.4	157	10.6	7.24	<0.01	33.7	3.76
42.5	42.0	1.49	118	7.15	156	5.10	9.80	0.50	35.5	3.11
73	43.1	0.12	117	7.10	146	12.5	4.89	0.61	35.0	2.39
103	46.3	0.41	114	7.80	143	8.00	4.99	0.17	35.5	5.50
133	43.2	2.53	120	9.34	143	5.34	0.185	<0.01	39.1	2.78
163	43.8	0.57	113	1.47	136	6.57	1.29	0.04	45.2	4.39

SD: standard deviation.

Acetate-Fe(II): buffered acetate extractable Fe(II); oxalate-Fe(III): acidic oxalate extractable Fe(III); BCD-Fe(III): BCD-extractable Fe(III); hydroxylamine-Fe(III): Hydroxylamine extractable Fe(III).

#### Acidic hydroxylamine extraction

This extraction was carried out following Lovley and Phillips [42] to estimate amorphous Fe(III) oxides. Under N_2_, 25 mL of mixing solution containing 0.25 M HCl and 0.25 M hydroxylamine was added to preweighed (∼1 g) frozen sediments in triplicate for a 1-h extraction while shaking. After centrifugation (4800 rpm) and filtration using polyether sulphone filtration membranes (0.22 µm, Membrana, Germany), supernatant was collected for Fe analysis. Another sub-sample was extracted by 0.25 M HCl solution (25 mL) by the same procedure as that described above. This extraction was used as a correction for H^+^-promoted Fe dissolution in the acidic hydroxylamine extraction. An analysis of a standard solution containing both Fe^2+^ and Fe^3+^ and the blank sample indicated that the filters did not interfere with Fe concentrations and speciation in filtered samples.

#### Acidic oxalate extraction

Acidic oxalate is capable of extracting Fe(II)-bearing minerals such as Fe(II) sulfides (pyrite excluded), Fe(II) carbonates (siderite and ankerite), amorphous and poorly crystalline Fe(III), and magnetite [20], [43]. Prior to determination of Fe(III) oxides, Fe(II) (pyrite excluded) was firstly dissolved by buffered acetate extraction, which left most Fe (oxyhydr)oxides relatively unaffected [20]. Under N_2_, 25 mL of NaAc-HAc buffer solution (pH 4.5) was added to preweighed frozen sediments (∼0.5 g) (in triplicate) for a 24-h extraction while shaking. After centrifugation, supernatants were filtered for Fe^2+^ analysis. Residual sediment pellets were water-washed twice and then subjected to a 6-h extraction by 0.2 M oxalate (pH 3.2) for determination of Fe(III) oxides. After centrifugation and filtration, supernatants were collected for Fe analysis.

#### Bicarbonate-citrate buffered sodium dithionite (BCD) extraction

The extraction procedure followed de Koff et al. [44], which is a modified version of Mehra and Jackson [45] and Lord [46]. Under N_2_, 20 mL of BCD solution (78 g/L sodium citrate, 9.3 g/L sodium bicarbonate, and 100 g/L sodium dithionite) was added to preweighed (∼1 g) frozen sediments in triplicate for a 2-h extraction in water bath at 75°C while shaking. After centrifugation, supernatants were filtered for Fe analysis. The BCD is capable of extracting Fe(II) sulfides (pyrite excluded), amorphous and crystalline Fe(III) oxides, but only a fraction of magnetite (<15%) [20], [47]. Fe(III) oxides extracted by the BCD were obtained as the difference between the BCD extraction and the buffered acetate extraction.

#### Magnetite extraction

Magnetite was separately estimated using the method of Poulton and Canfield [20] with a minor modification, that is, preweighed frozen sediments were sequentially extracted by BCD (2 h) at 75 °C and then by acidic oxalate (6 h), the oxalate-extractable Fe is an estimate of magnetite.

All extracted Fe was determined by colorimetry [48]. Variability (i.e., percent difference) among triplicates was 0.1−6.2% (mean 1.8%) for the buffered acetate extraction, <0.1−12.5% (mean 3.9%) for the acidic hydroxylamine extraction, 0.1−12.6% (mean 6.8%) for the acidic oxalate extraction, 0−11.2% (mean 3.8%) for the BCD extractions, and 7.4−16.0% (mean 10.1%) for the magnetite extractions.

### Kinetic experiments

Kinetic experiments of Fe(III) oxide dissolution were undertaken at room temperature using ascorbic acid as a reductant at pH 3.0 and 7.5, respectively, by monitoring time-dependent Fe^2+^ release from the sediments. The experimental procedure at pH 3.0 was similar to Postma [22] and Larsen et al. [21]. Briefly, a preweighed frozen sediment (∼4 g) was loaded into a reactor containing 1 L deoxygenated ascorbic acid solution (10 mM), which was pre-adjusted to pH 3.0 using dilute HCl. After being tightly sealed with a rubber stopper, the reactor was magnetically stirred and purged with N_2_ during 12−27-h experiments to ensure a homogeneous and anoxic system. Fitted through the stopper were three outlets, one for sampling and the two others for gas in/out, and a pH electrode for pH monitoring. The pH was stabilized at 3.0 by adding dilute HCl with an autotitrator. The suspension was periodically sampled using 1-mL syringes and filtered (0.22 µm) directly into glass vials containing Na-acetate-buffered ferrozine solution for Fe determination by colorimetry [48]. At pH 3.0, H^+^-promoted dissolution of Fe(II)-bearing minerals such as Fe(II) monosulfides and siderite (FeCO_3_) is significant, while dissolution of Fe(III) oxides is negligible [23]. Thus kinetic dissolution in HCl solution (pH 3.0) was also carried out on another subsample as the correction for H^+^-promoted Fe(II) dissolution in the ascorbic acid solution.

The experimental procedure at pH 7.5 was the same as that described above with the exception that the reductant was CB-buffered ascorbate (pH 7.5) [24], [28]. In this case, H^+^-promoted dissolution of Fe(II)-bearing minerals was negligible, and thus a correction was not needed.

Optimized values of *m*_0_, *k*′ and *γ* were simultaneously obtained by fitting the kinetic data to Eq. (4) using a nonlinear least squares procedure (Origin ver. 8.5). In what follows, where a discrimination is necessary, *m*_0_, *k*′ and *γ* obtained at pH 3.0 are denoted as *m*_0_(3.0), *k*′(3.0) and *γ*(3.0), and the ones obtained at pH 7.5 as *m*_0_(7.5), *k*′(7.5) and *γ*(7.5).

Note that not all the runs of the kinetic experiments were replicated due to tremendously huge workload. Only two selected runs at pH 3.0 and 7.5, respectively, were performed in duplicate to examine the reproducibility of the experiments. Results showed that variability (i.e., percent difference) for dissolved Fe^2+^ (i.e., *m*_t_) between duplicates of the selected runs was less than 6%, however, *m*_0_, *k*′ and *γ* had larger variability (7−10%) due to error accumulation in the fittings.

## Results

### Pool sizes of selective extractions

**Figure 2:**
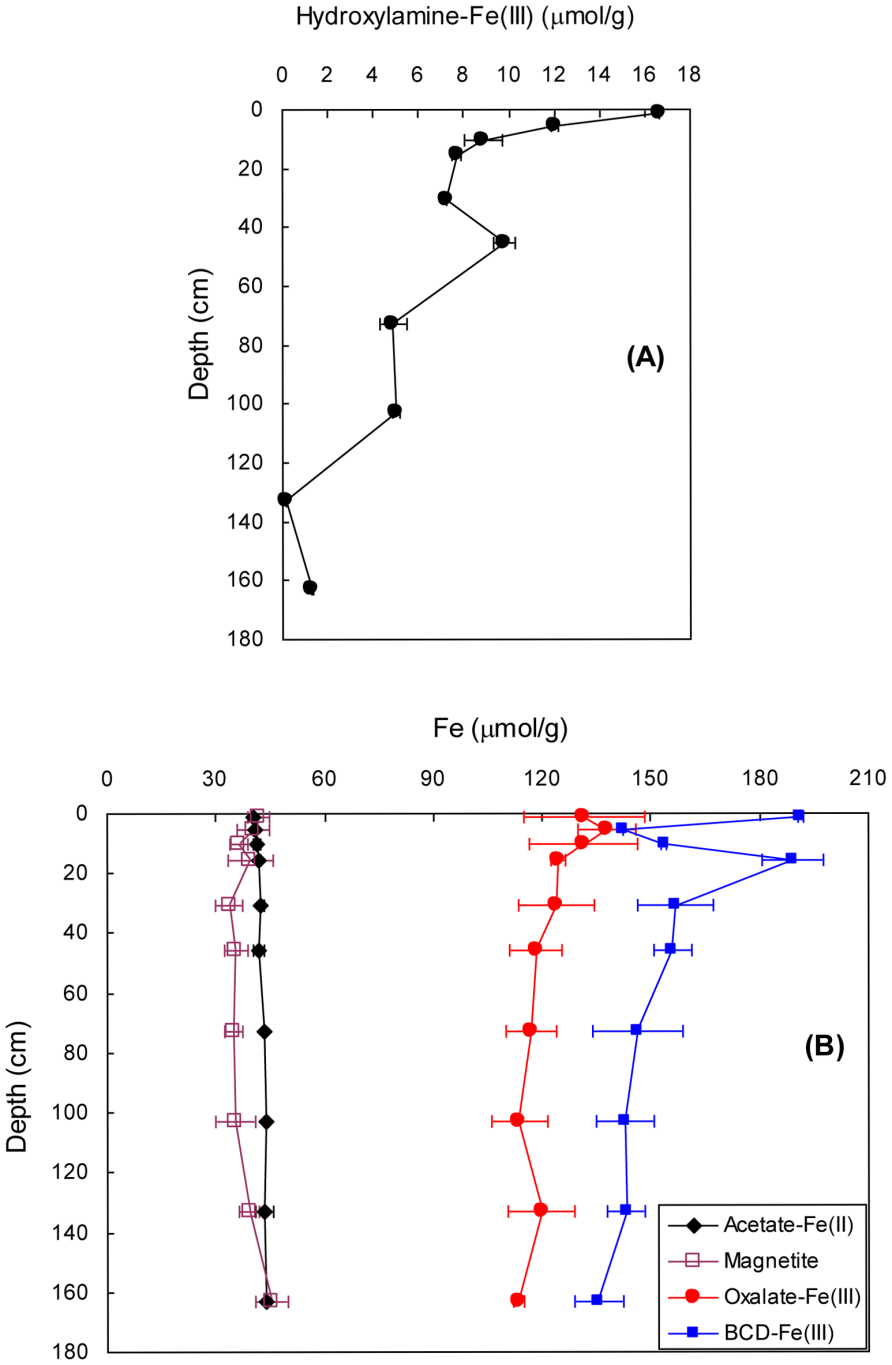
Depth profiles of extracted Fe (µmol per g dry sediment). (A) hydroxylamine-extractable ferric Fe (hydroxylamine-Fe(III)), (B) oxalate-extractable ferric Fe(III) (oxalate-Fe(III)), BCD-extractable ferric Fe (BCD-Fe(III)), buffered-acetate extractable ferrous Fe (acetate-Fe(II)), and magnetite. Error bars represent ±1 standard deviation.

The sizes of Fe(II) and Fe(III) pools are shown in Table 1 and Fig. 2. The hydroxylamine reducible Fe(III) oxides displayed a quick downcore decrease from 16.7 to 7.7 µmol/g in the upper 16 cm, and then a gradual decrease to very low values (0.19−1.3 µmol/g) below a depth of 130 cm. The oxalate extractable Fe(III) pool was in the range of 113−138 µmol/g and displayed an apparent downcore decrease in the upper 16 cm, below which only a small decrease was observed. The BCD-extractable Fe(III) pool was in the range of 136−191 µmol/g and exhibited a quick downcore decrease in the upper 11 cm. A peak value of the pool occurred at around 16-cm depth, below which a small decrease with depth was observed. Magnetite contents were in the range of 33.7−45.2 µmol/g with no clear depth variability. The average (38.3 µmol/g) is slightly lower than that for the ECS surface sediments (46.4 µmol/g) [35].

### Kinetic parameters at pH 3.0 and 7.5

**Figure 3:**
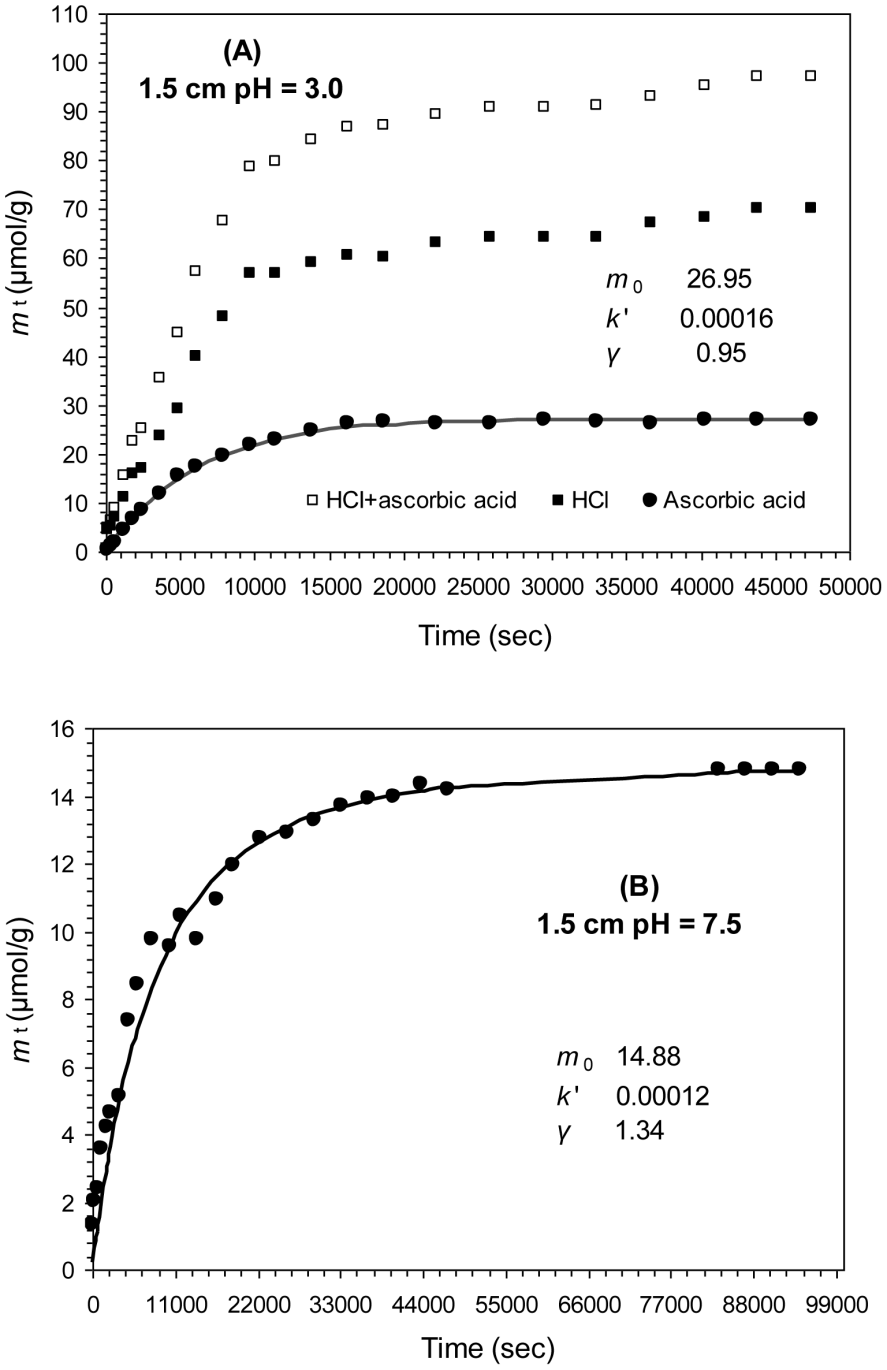
Representative curves of time-dependent release of Fe^2+^ from sediments (1.5 cm depth). (A) Fe^2+^ release in HCl solution at pH 3.0 (▪) and in HCl + ascorbic acid solution at pH 3.0 (□). Fe^2+^ release by reductive dissolution (•) is obtained as the difference between the release in HCl + ascorbic acid solution and in HCl solution. (B) Fe^2+^ release in buffered ascorbate (pH 7.5). Solid lines in (A) and (B) are nonlinear least squares fits to equation 4.

**Figure 4:**
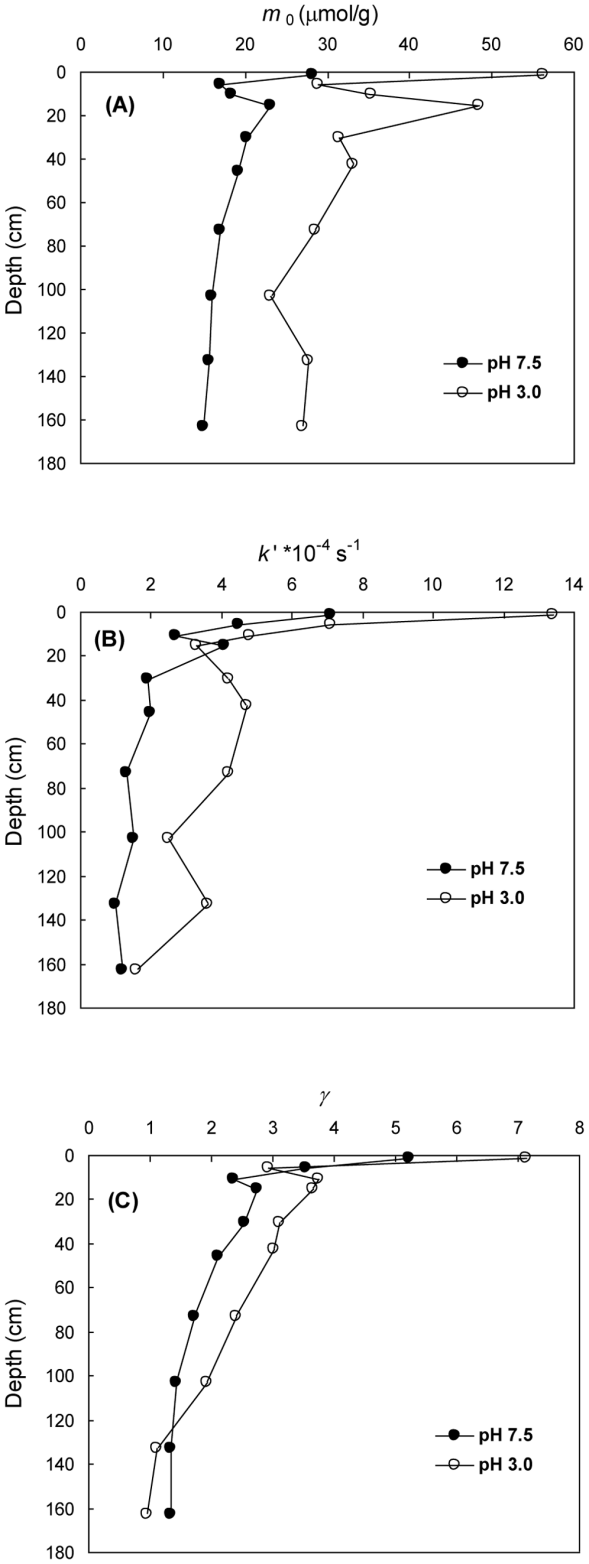
Depth profiles of *m*_0_, *k*′ and *γ* at pH 3.0 and 7.5. (A) *m*_0_, (B) *k*′, (C) *γ*.

Representative curves of time-dependent Fe^2+^ release from the sediments at pH 3.0 and 7.5 are shown in Fig. 3. Optimized values of *m*_0_, *k*′ and *γ* are shown in Fig. 4. The values of *m*_0_(3.0), *k*′(3.0) and *γ*(3.0) were 23−57 µmol/g, 1.6−13.4×10^−4^ s^−1^, and 0.95−7.13, respectively. The values of *m*_0_(7.5), *k*′(7.5) and *γ*(7.5) were 14−29 µmol/g, 1−1.7×10^−4^ s^−1^, and 1.33−5.22, respectively. Although the *m*_0_, *k*′ and *γ* at pH 3.0 were larger than the corresponding values at pH 7.5, the three kinetic parameters at the two pHs displayed similar depth-dependent patterns. For example, all of the kinetic parameters exhibited maximum values in the surface sediments, a rapid decrease with depth to 16 cm, followed by a more gradual decline below 16 cm.

## Discussion

### Fe speciation by selective extractions

#### Hydroxylamine reducible Fe(III)

Under acidic conditions, 1-h hydroxylamine extraction mainly targets amorphous Fe(III) oxides, and the extraction provides a simple and rapid technique to assess the availability of Fe(III) oxides for microbial reduction, albeit being underestimated [42]. The maximum of hydroxylamine reducible Fe(III) (16.7 µmol/g) in the surface sediments of the current study is comparable to the values for surface sediments in the Potomac Estuary (<20 µmol/g) [42], though the site of the current study has higher salinity (34−35‰) and sulfate concentration (26−28 mM) than the Potomac Estuary (salinity: 11‰, sulfate: 10−15 mM). The rapid downcore decrease in the hydroxylamine reducible Fe(III) in the upper 16 cm indicates a quick consumption through reduction. Although a variety of Fe(III) oxides, including poorly reactive sheet silicate Fe(III), are chemically and microbially reducible on wide timescales, highly reactive Fe(III) oxides, particularly amorphous Fe(III) phases, are usually consumed preferentially. The presence of acid volatile sulfides and pyrite (FeS_2_) in the inner shelf sediments of the ECS suggests that chemical reduction has played an important role in Fe(III) reduction [35], [37], [40], [49], while dissimilatory Fe(III) reduction may have also played a role, according to previous biogeochemical and microbiological observations [35], [39]-[41]. The persistence of hydroxylamine reducible Fe(III) to a depth of around 100 cm in this study, albeit being at a low level, indicates that amorphous Fe(III) phases was not depleted over this depth interval. This is quite different from the findings of Lovley and Phillips [42], who found that hydroxylamine reducible Fe(III) in the Potomac Estuary sediments decreased rapidly with depth and became nearly exhausted below the upper 3 cm. The persistence of the Fe(III) pool at the deeper sediments of this study can be ascribed to small consumption of the pool by reaction with limited hydrogen sulfide. Limited availability of hydrogen sulfide in porewater of the sediments has been confirmed by previous studies, which suggested that sulfate reduction and thus sulfide production in the ECS shelf sediments was limited by generally low degradability of OM and high redox potential caused by intensive physical reworking [37], [40], [49], [50]. In the deeper layers, microbial Fe(III) reduction is expected to be insignificant, if any, because high activity of iron reducers is generally restricted to the upper suboxic/anoxic layers.

#### Acidic oxalate- and BCD-extractable Fe

In the present study, all Fe(II) sulfides (pyrite excluded) were extracted by buffered acetate prior to the oxalate extraction. Thus oxalate-extractable Fe can be used as an estimate of amorphous and poorly crystalline Fe(III) oxides plus magnetite. Magnetite is usually a minor mineral in modern marine sediments, but our previous study indicated that the ECS surface sediments have magnetite contents (25−74 µmol/g, with an outlier of 104 µmol/g) [35] higher than for many other marine sediments (<17.8 µmol/g) [51], [52]. Magnetite contents in the core studied (33.7−45.2 µmol/g) are within the range for the ECS surface sediments [35].

The amounts of amorphous and poorly crystalline Fe(III) oxides can be estimated by subtracting magnetite content from the oxalate extraction. The estimated amounts of amorphous and poorly crystalline Fe(III) oxides after correction for the magnetite were in the range of 68−98 µmol/g. Amorphous and poorly crystalline Fe(III) oxides are the major components of the Fe(III) pool responsible for quick buffering of dissolved sulfide in porewater [53], [54]. The high amounts of this pool over the entire core imply that it has not been substantially consumed and is readily available for further buffering of dissolved sulfide. This is in agreement with our previous findings [40], [50].

Contents of crystalline Fe(III) oxides (mainly goethite, akaganéite and hematite) and the total crystalline Fe oxides including magnetite can be estimated using the results of the BCD extractions, the oxalate extractions, and the magnetite. Estimated contents of crystalline Fe(III) oxides were 45–104 µmol/g and the total contents of crystalline Fe oxides including magnetite were 85−144 µmol/g. Both the BCD- and oxalate-extractable Fe(III) oxides displayed only a small downcore decrease and maintained high values at depth, suggesting that there has been only a small consumption of the two Fe(III) pools during long-term diagenesis. A subsurface (∼16 cm depth) peak of the BCD-extractable Fe(III) is probably due to accumulation of some well crystalline Fe oxides at the redox interface due to aging of newly generated Fe(III) oxide phases given that oxalate-extractable Fe(III), mainly amorphous and poor crystalline Fe(III) oxides and magnetite, did not display a peak at similar depths.

### Kinetic characterization of diagenetic effects on Fe(III) oxide reactivity

As shown in Fig. 4, depth-dependent variability of *m*_0_, *k*′ and *γ* at both pH 3.0 and 7.5 indicate that diagenesis has exerted a great impact on reactivity of Fe(III) oxides in the uppermost layer while a much less but persisting impact at depth. Maximum *m*_0_, *k*′ and *γ* occurring in the surface/subsurface layers is not unexpected because regeneration of highly reactive Fe(III) oxides by reoxidation of Fe^2+^ in the layers, particularly at the redox interface, is always active; on the other hand, intensive physical reworking causes mixing of surface/subsurface sediments and aging of Fe(III) oxides to varying extents due to repetitive resuspension, which is expected to render Fe(III) oxide assemblages enormously heterogeneous. In the ECS inner shelf, highly dynamic fluidized mud at the surface is expected to encourage upward diffusion and subsequent reoxidation of Fe^2+^ in the subsurface and surface sediments, as well as mixing and aging of Fe(III) oxides [49], [50], [56]. A similar phenomenon was also observed for manganese oxides in the North Sea sediments [55]. In addition, bioturbation by macrofauna may create diverse microenvironments and re-distribute sediments and Fe(III) oxides [49], further enhancing the heterogeneity of Fe(III) oxide reactivity. The quick downcore decrease in *m*_0_, *k*′ and *γ* in the uppermost zone indicates preferential consumption of highly reactive Fe(III) oxides by chemical and/or microbial reduction, leaving more resistant and less heterogeneous residual Fe(III) phases behind. This is in agreement with the accepted notion that highly reactive Fe(III) oxides exhibit a quick downcore decrease in anoxic sediments and also in agreement with the result of the rapid downcore decrease in amorphous Fe(III) oxides quantified by the hydroxylamine extraction (Fig. 2). It should be pointed out that the reduction of Fe(III) oxides is not necessarily reflected in the profile of buffered-acetate extractable Fe(II) (Fig. 2B) because a large fraction of Fe(II) occurs as pyrite, which is non-extractable by buffered acetate.

**Figure 5:**
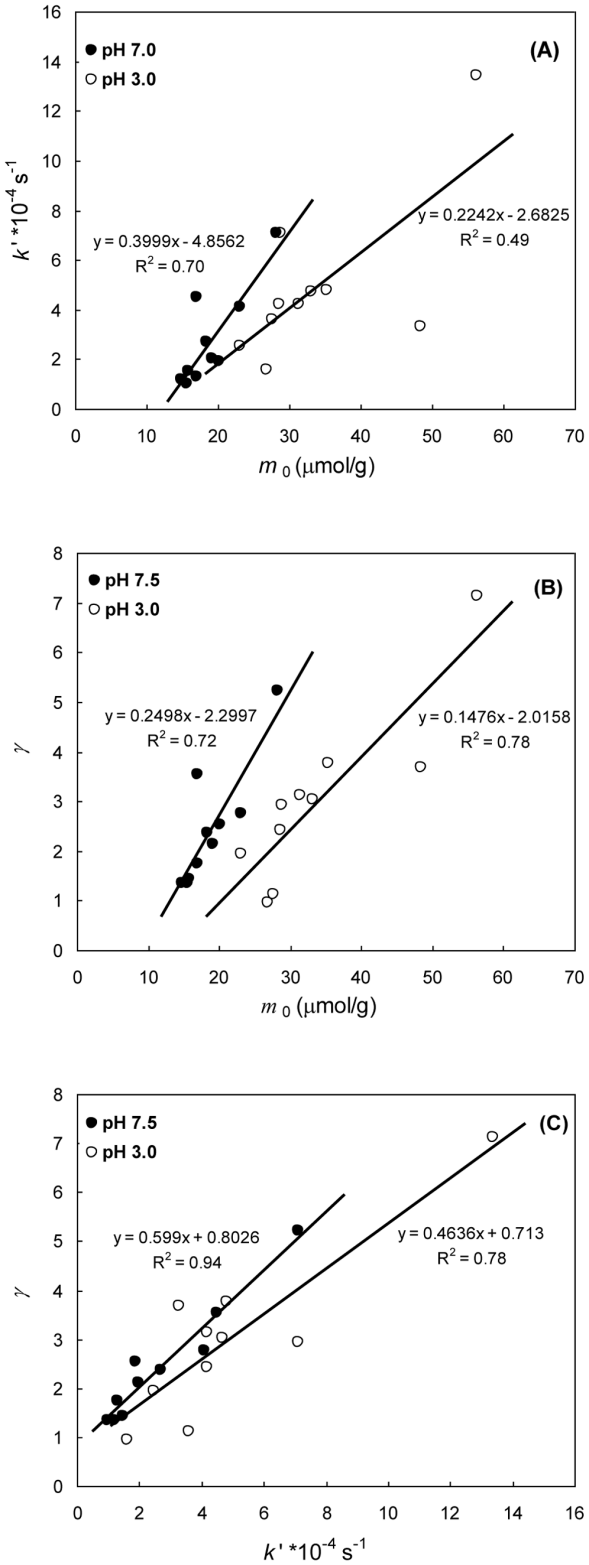
Correlations of *m*_0_ vs. *k*′ (A), *m*_0_ vs. *γ* (B), and *k*′ vs. *γ* (C).

The slow decrease in *m*_0_ from 16 cm depth to the bottom indicates that the Fe(III) oxide pool has been subjected to a slow but persisting reduction over a long timescale, which can be ascribed to a combined result of progressive decrease in Fe(III) oxide reactivity and limited availability of dissolved sulfide to react with the Fe(III) pools. The slow decrease in *m*_0_ also resulted in a concomitant decrease in *k*′ and *γ*. Coupling among the three kinetic parameters is illustrated by generally good linear correlations (*t* test, *p*<0.05) among them (except a weak correlation between *m*_0_(3.0) and *k*′(3.0)) (Fig. 5). The coupling is expected to be the result of diagenesis as the common driving force. Actually, it is hard to imagine that a similar coupling does occur in sediments where reactive Fe does not share the common origins and driving forces in cycling. It should be pointed out, however, that a similar coupling may not be observed in a short sediment core, and common diagenesis does not necessarily result in a linear coupling among the three parameters when some specific highly reactive Fe(III)-bearing minerals dominate the sediments. For example, a rapid downcore decrease in *m*_0_(7.5) in the subsurface layers of the Appels and Waarde sediments and a gradual decrease in the deeper layers (35−41 cm) were observed, however, *k*′(7.5) and *γ*(7.5) displayed no clear depth trend over the entire depth interval, due, at least, to abundant but highly varying contents of authigenic Fe(III)-P minerals, a highly reactive Fe(III)-bearing phase [24], [28].

In comparison with a wide range of initial rates *k*′(3.0) reported for Fe(III) oxide coated sands, soils, riverine and marine sediments (10^−7^−10^−2^ s^−1^) [21], [22], [25]–[27], [57], the change in the *k*′(3.0) in the present study is small (1.6−13.4×10^−4^ s^−1^). Also the *k*′(7.5) values are in a small range (1−1.7×10^−4^ s^−1^) and generally lower than the values for the Appels and Waarde sediments bearing highly reactive authigenic Fe(III)-phosphate minerals (4.0−19.3×10^−4^ s^−1^) at the corresponding depths [24], [28]. The range of the *γ*(3.0) (0.95−7.13) in the present study is larger than that reported in the literature (0.79−5.14) [21], [22], [25]–[27], [57], while the *γ*(7.5) (1.33−5.22) is in a narrower range than that reported in the literature (*γ*(7.5) = 1.1−10) [24], [28].

**Figure 6:**
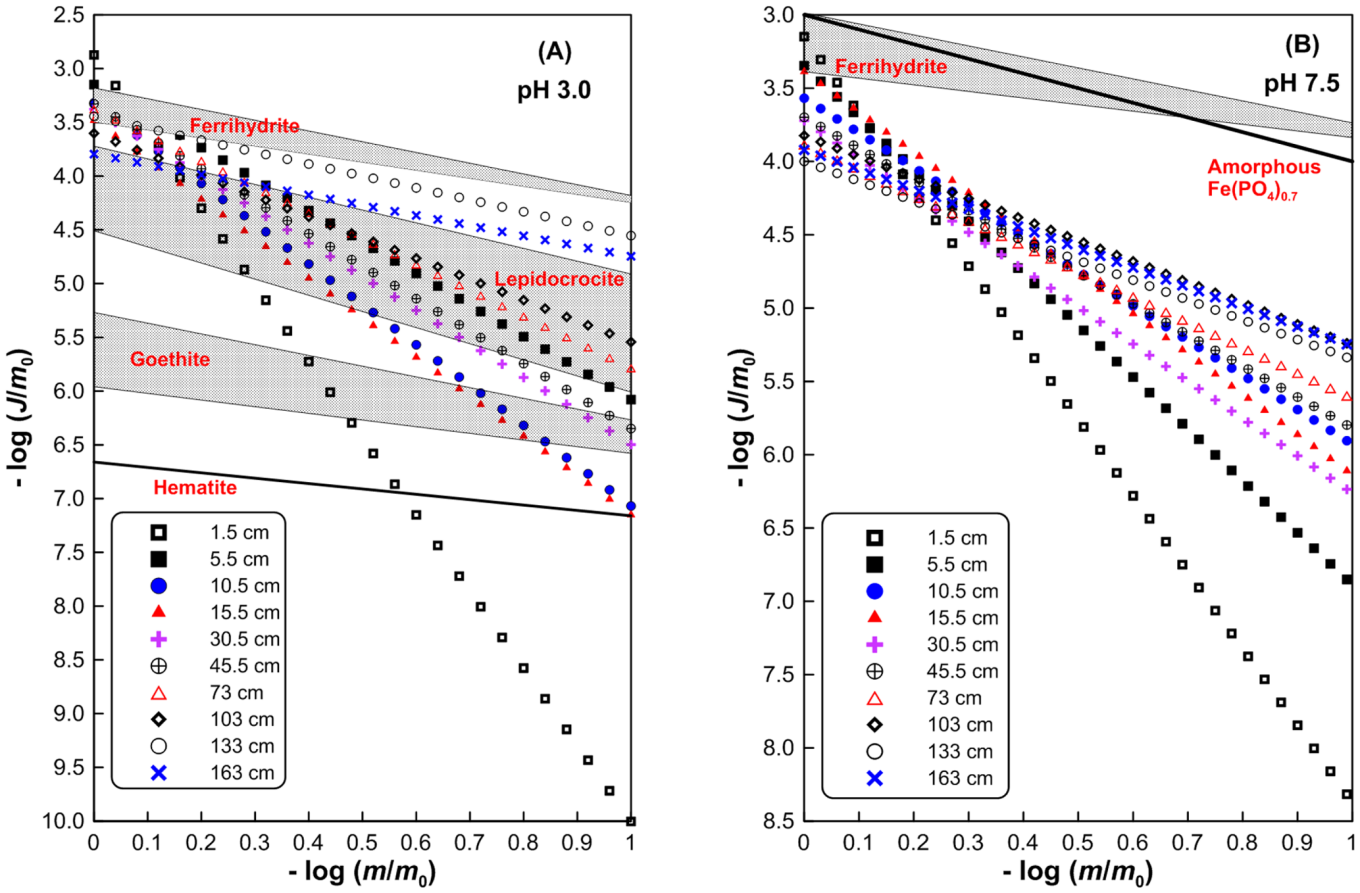
Reactivity of sedimentary Fe(III) oxides as compared to that of synthetic Fe(III) oxides. (A) Reduction rates at pH 3.0 normalized over initial mass (*J*/*m*_0_) versus the fraction (*m*/*m*_0_) remaining in the solid phase. Reactivity of ferrihydrite, lepidocrocite, goethite, and hematite at pH 3.0 is from Larsen and Postma [23] and Pedersen et al. [63], [64]. (B) Reduction rates at pH 7.5 normalized over initial mass (*J*/*m*_0_) versus the fraction (*m*/*m*_0_) remaining in the solid phase. Reactivity of amorphous Fe(PO_4_)_0.7_ and ferrihydrite at pH 7.5 is from Hyacinthe and van Cappellen [28] and Raiswell et al. [29], respectively. Gray shadings in (A) and (B) delineate the ranges of Fe(III) oxide reactivity.

The plots of −log(*m*/*m*_0_) versus −log(*J*/*m*_0_) (Fig. 6) can illustrate temporal evolution of dissolution rate as Fe(III) oxide dissolution is in progress [21], [22]. Figure 6 can also provide some mineralogical information of Fe(III) oxides, in a relative sense, by comparing with the reactivity of some synthetic Fe(III) oxides. For this purpose, the reactivity of some synthetic Fe(III) oxides is also shown in Fig. 6. At pH 7.5, only ferrihydrite and amorphous Fe(PO_4_)_0.7_ (Fig. 6B) are shown because they are the only synthetic phases whose reactivity at this pH is available [24], [29]. The *k*′(pH 3.0) in the uppermost sediments is slightly higher than that for synthetic ferrihydrite while the *k*′(pH 3.0) in the deeper sediments is between that for synthetic ferrihydrite and lepidocrocite. This indicates that Fe(III) oxides in the uppermost sediments have higher reactivity than synthetic ferrihydrite. In the deeper layers, however, the reactivity decreased to be lower than for synthetic ferrihydrite but still higher than for lepidocrocite. The *k*′(7.5) values over the upper 16 cm (with the exception at 10.5 cm depth) are within the range for synthetic ferrihydrite (aging for 3 to 11 days), while the *k*′(7.5) in the deeper sediments is much lower than that for ferrihydrite. Over the entire depth interval the *k*′(7.5) is lower than that for amorphous Fe(PO_4_)_0.7_. It should be pointed out that the reactivity of the Fe(III) oxide assemblages is 2−3 orders of magnitude higher than that for synthetic ferrihydrite that has been subjected to de-watering and aging to varying extents (*k*′(7.5) = 0.3−4.7×10^−6^ s^−1^) [29].

After 90% (i.e., −log(*m*/*m*_0_) = 1) of the Fe(III) oxides in the upper sediments has dissolved, dissolution rates of the residual phases at pH 3.0 and 7.5 decreased by 7 and 5 orders of magnitude, respectively, relative to their corresponding initial rates. This is due to great heterogeneity of the Fe(III) oxide assemblages, comprising both highly reactive and more resistant phases. Similarly, Van der Zee and van Raaphorst [55] found a change up to 6 and 9 orders of magnitude for manganese oxides in moderately energetic Frisian Front and highly energetic German Bight sediments, respectively. Quick and preferential consumption of highly reactive Fe(III) oxides has left much more resistant and less heterogeneous phases (with reactivity much more resistant than or comparable to that of hematite) behind. As a result, in the deeper sediments, the decrease in dissolution rates became generally smaller relative to their initial rates. Although there is only a small variation in the initial rates (i.e., *k*′) among the sediments at different layers, differentiating decreases in reactivity due to incongruent dissolution have resulted in a huge discrepancy (up to five and three orders of magnitude at pH 3.0 and 7.5, respectively) in dissolution rates of the residual Fe(III) oxides after dissolution of 90%. This suggests that heterogeneity has exerted a significant impact on temporal evolution of the dissolution process and dissolution rates at the later stage. This behavior may be important in controlling Fe(III) oxide dissolution in natural anoxic sediments where Fe(III) oxide reduction has proceeded to a great extent. It is worthy to point out that, the occurrence of *m*_0_, *k*′ and *γ* peaks at ∼16 cm depth (with the exception of *k*′(3.0)) are probably the result of repetitive oxidation and reduction of Fe at the redox interface, which is seemingly supported by the fact that the *m*_0_ peaks are roughly corresponding to the BCD-extractable Fe(III) peak (Fig. 2).

### Selective extractions versus kinetic dissolution

#### Selective extractions versus *m_0_*(3.0)

**Figure 7:**
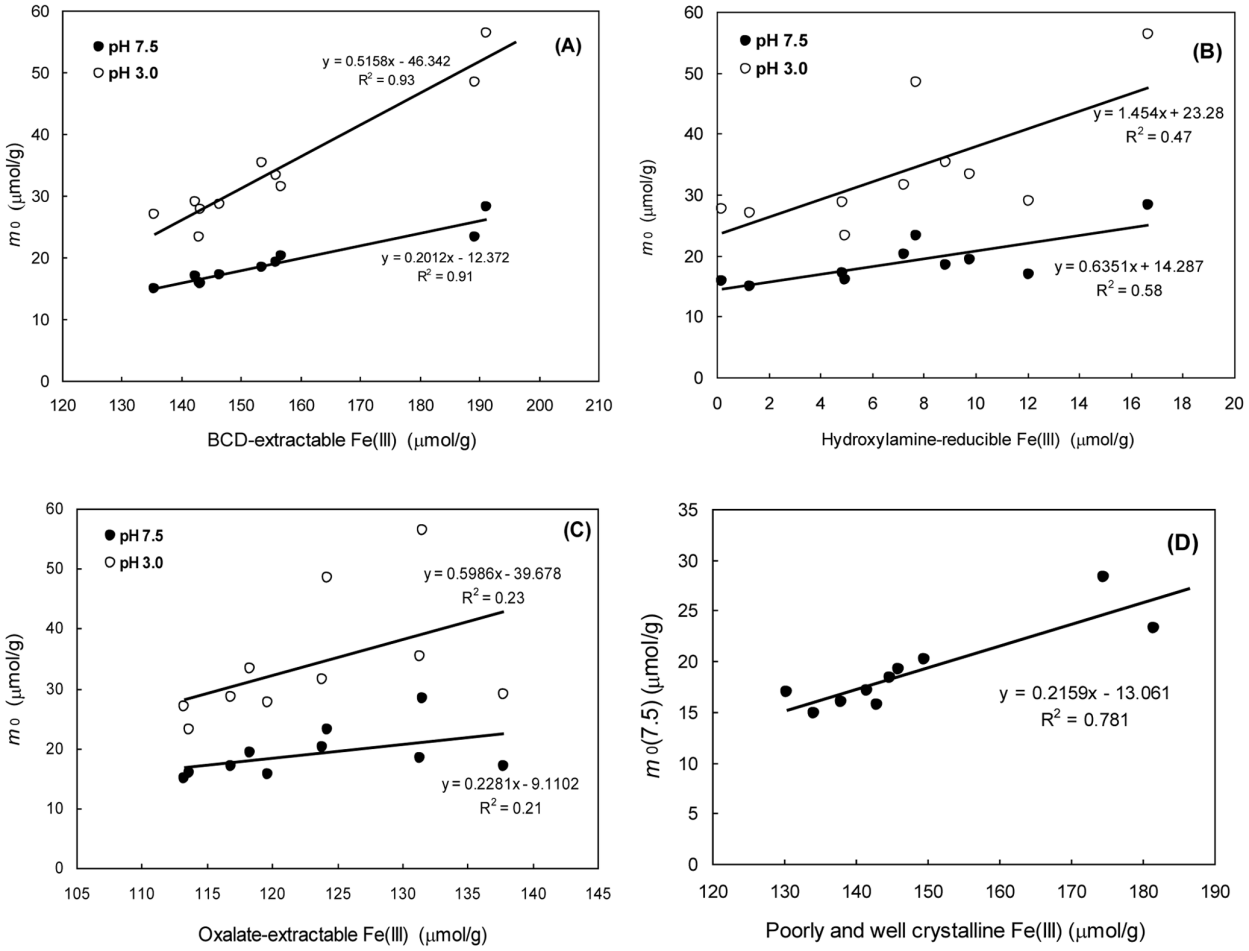
Correlations of extracted Fe(III) vs. three kinetic parameters. (A) BCD-extractable Fe(III) vs. *m*_0_ at pH 3.0 and 7.5; (B) hydroxylamine-extractable Fe(III) vs. *m*_0_ at pH 3.0 and 7.5; (C) oxalate-extractable Fe(III) vs. *m*_0_ at pH 3.0 and 7.5; (D) poorly and well crystalline Fe(III) vs. *m*_0_ at pH 7.5.

Both selective extraction and the reactive continuum model have been used for characterization of the reactivity of Fe(III) oxides, however, to compare the two techniques is not straightforward because the former reflects separate measurements while the kinetic parameters are derived from time-dependent experiments. Larsen et al. [21] found that *m*_0_(3.0) was only slightly higher than oxalate extractable Fe(III) but certainly lower than BCD-extractable Fe(III) in a shallow sandy aquifer (Rømø, Denmark), and thus concluded that most of the heterogeneity fell within the fraction extracted by oxalate. In the current study, the *m*_0_(3.0) is much lower than both the BCD- and oxalate-extractable Fe(III) but nearly two-fold the hydroxylamine extractable Fe(III). This suggests that the *m*_0_(3.0) cannot be quantitatively linked to any of the three selective extractions. Note that a strong linear correlation between the *m*_0_(3.0) and the BCD-extractable Fe(III) (*t* test, *p*<0.05) (Fig. 7A) may suggest that the *m*_0_(3.0) represents Fe(III) oxide assemblages spanning a wide spectrum of mineralogical phases from amorphous to crystalline Fe(III) oxides. This is probably the reason for the weak dependence between the *m*_0_(3.0) and the amorphous Fe(III) pool quantified by the hydroxylamine extraction (Fig. 7B). A very weak linear correlation between the *m*_0_(3.0) and the oxalate-extractable Fe(III) (*t* test, *p*<0.05) (Fig. 7C) is probably due to the fact that acidic oxalate can effectively dissolve magnetite but ascorbic acid at pH 3.0 cannot. In Larsen et al. [21], the rough similarity between the *m*_0_(3.0) and the oxalate-extractable Fe(III) is probably only a site-specific feature given that oxalate-extractable Fe(III) (<3 µmol/g) in the aquifer sediments was much lower than in marine sediments [43], [58].

#### Selective extractions vs. *m_0_*(7.5)

The *m*_0_(7.5) can be used as a maximum estimate of bioavailable Fe(III) present in sediments [24], [28]. Hydroxylamine-extractable Fe(III) has also been used to assess the availability of Fe(III) oxides to microbial reduction, albeit being an underestimate to some extents [42]. In the present study, the hydroxylamine-extractable Fe(III) is 60−71% of the *m*_0_(7.5) in the surface/subsurface layers but decreases to 8−12% at the bottom. The larger *m*_0_(7.5) values than amorphous Fe(III) oxides quantified by the acidic hydroxylamine extraction imply that the maximum bioavailable Fe(III) (i.e., *m*_0_(7.5)) should include both amorphous and other less reactive Fe(III) phases, which is seemingly supported by strong linear correlations of the *m*_0_(7.5) vs. both the BCD-extractable Fe(III) (Fig. 7A) and the amounts of poorly and well crystalline Fe(III) oxides (i.e., the difference between the BCD extraction and the hydroxylamine extraction) (*t* test, *p*<0.05) (Fig. 7D). This is in agreement with the findings that although amorphous Fe(III) oxides are always more readily susceptible to microbial reduction, iron reducers are also capable of reducing other less reactive Fe(III) oxides, even poorly reactive sheet silicate Fe(III) [59], [60]. Similar to the case at pH 3.0, there is only a weak correlation between the *m*_0_(7.5) and the hydroxylamine- and oxalate-extractable Fe(III) (*t* test, *p*<0.05), respectively. Based on the analysis above, it appears that the reasons for the weak correlations between the *m*_0_(3.0) and the hydroxylamine- and oxalate-extractable Fe(III) are applicable as well to the weak correlations between the *m*_0_(7.5) and the two Fe(III) pools.

The *k*′(7.5) can be used as a forecast proxy of maximum Fe(III) reduction rate per cell (*ν*_max_) based on strong linear correlation of log *k*′(7.5) vs. log*ν*_max_ [24]. However, the *k*′(7.5) probably cannot directly represent the *in situ* macroscopic reactivity and initial rate of microbial Fe(III) oxide reduction in incubating and natural conditions because, by fitting available literature data of microbial Fe(III) oxide reduction to the reactive continuum model (Eq. 1), Davranche et al. [61] found that the *k*′ was in the range of 2.2×10^−7^−1.85×10^−5^ s^−1^, much lower than the *k*′(7.5) in the present study, and the *γ* was in the range of 5.77−44.5, higher than the present *γ*(7.5). Great rate deceleration (i.e., large *γ*) while microbial reduction progresses in incubating and natural conditions, according to Roden [27], [62], is due to adsorption of reaction products and secondary mineral formation that lead to a progressive inhibition of the reduction. In addition, microbial reduction also exhibits very large *γ* in incubations which are limited by bacterial cell number [61].

It should be pointed out that only one site was used for this study, and thus spatial heterogeneity of Fe(III) oxide reactivity cannot be revealed. However, this study may have depicted a general feature of the entire inner shelf since the inner shelf (except the Yangtze Estuary) has many things in common including hydrodynamic conditions and main sediment sources. This argument is seemingly supported by generally similar diagenesis of Fe and sulfur in the northern and southern inner shelves [36], [37], [40], [49].

## Summary and Conclusion

Amorphous Fe(III) oxides were quickly consumed in the upper layer of the ECS shelf sediments but not completely depleted until at least 100-cm depth due to low availability of hydrogen sulfide towards reaction with the pool. The persistence of amorphous and poorly crystalline Fe(III) oxide pools suggests that they are readily available for quick buffering of dissolved sulfide in the sediments.

Downcore decreases in *m*_0_, *k*′ and *γ*, much quicker in the uppermost layer than at depth, indicate simultaneous decreases in content, reactivity and heterogeneity of reactive Fe(III) oxides, resulting in good linear correlations among the three kinetic parameters (except a weak correlation of *m*_0_(3.0) vs. *k*′(3.0)). Initial rates among the sediments at different depths are in a small range (within one order of magnitude), while time-dependent dissolution rate is quite different as the dissolution is in progress, which is strongly dependent on heterogeneity of Fe(III) oxides. For example, the discrepancies can reach five and three orders of magnitude at pH 3.0 and 7.5, respectively, after 90% of the Fe(III) oxides has dissolved. This behavior cannot be revealed by traditional selective extraction.

None of the three extractable Fe(III) pools is quantitatively comparable to the *m*_0_(3.0), while a strong linear correlation of the *m*_0_(3.0) vs. the BCD-extractable Fe(III) suggests that the *m*_0_(3.0) may represent Fe(III) oxide assemblages spanning amorphous and crystalline phases. Much lower amorphous Fe(III) than microbially available Fe(III) predicted by the *m*_0_(7.5) and strong linear correlations of the *m*_0_(7.5) vs. the pool of poorly and well crystalline Fe(III) oxides suggest that maximum bioavailable Fe(III) may include both amorphous and other less reactive Fe(III) phases. Although the *k*′(7.5) can be used as a forecast proxy of maximum Fe(III) reduction rate per cell (*ν*_max_), it cannot directly represent the *in situ* macroscopic reactivity and initial rate of microbial Fe(III) oxide reduction in natural conditions.
